# The addition of sweetness reduces aversion to high salt concentrations in patients with chronic kidney disease

**DOI:** 10.1038/s41598-025-09602-x

**Published:** 2025-07-07

**Authors:** Natsuko Okuno-Ozeki, Yusuke Kohama, Hiromu Taguchi, Yuka Kawate, Keiichi Tamagaki, Takuya Taniguchi, Takashi Hirao, Satoaki Matoba, Tetsuro Kusaba

**Affiliations:** 1 Department of Nephrology, Graduate School of Medical Science, Kyoto Prefectural University of Medicine, 465 Kajii-cho, Kamigyo-ku, Kyoto, 602-8566 Japan; 2 Research and Development Headquarters, House Foods Group Inc., Chiba, Japan; 3 Department of Food Sciences and Nutrition, School of Food Sciences and Nutrition, Mukogawa Women’s University, Hyogo, Japan; 4 Department of Cardiovascular Medicine, Graduate School of Medical Science, Kyoto Prefectural University of Medicine, Kyoto, Japan

**Keywords:** Taste interactions, Taste test, Salt taste, Sweet taste, Aversion threshold, Chronic kidney disease, Chronic kidney disease, Nutrition

## Abstract

Reducing salt intake is crucial for managing hypertension. While efforts to reduce salt intake are based on the assumption that people have a preference for salt, it is also important to acknowledge that there is an aversion to high levels of salt. A recent study has highlighted that not only the taste recognition but also the aversion to high salt were altered in CKD patients. However, the effect of combining various tastes on this aversion is still unclear. This study examined how adding sweetness affects salt aversion in CKD patients. A taste test was conducted on 100 healthy volunteers and 66 CKD patients using filter paper impregnated with varying concentrations of NaCl, sucrose, tartaric acid, and quinine hydrochloride. The aversion threshold was defined as the lowest concentration perceived as unpleasant. We measured aversion thresholds for combinations of sweet with salty, sour, and bitter tastes. Aversion to salt, sour, and bitter tastes increased with concentration. However, 44% of healthy subjects and 79% of CKD patients showed no aversion to high salt. Adding sweetness significantly reduced salt aversion, with 52% of healthy subjects and 92.4% of CKD patients showing no aversion to high salt. Sweetness slightly reduced aversion to sour tastes but had no effect on bitter taste in either group. Sweetness reduces aversion to high salt in both healthy individuals and CKD patients. To limit salt intake, not only should salt content be reduced, but excessive sweetness should also be avoided, as it lowers salt aversion.

**Supplementary Information:**

The online version contains supplementary material available at 10.1038/s41598-025-09602-x.

## Introduction

With the aging societies, the number of chronic kidney disease (CKD) patients is increasing worldwide^1^. Hypertension is one of the most important risk factors for the development of renal disease, and proper blood pressure control is important not only for the development of renal disease but also for the prevention of cardiovascular disease^2,3^. Reducing salt intake is important for proper blood pressure control, and various hypertension guidelines, renal disease guidelines, and WHO recommend limiting salt intake to 5 g/day or less as part of dietary therapy^3–6^. Although various approaches to salt reduction have been proposed, the recommended salt intake level has not been achieved^7^. Dietary taste preferences and individual taste sensitivities are thought to strongly influence salt intake, which may make reducing salt intake more difficult.

Salt restriction is generally based on the assumption that people prefer salt; however, animal experiments have shown that mammals prefer low concentrations of salt but show an aversive response to high concentrations of salt^8–10^. As for human studies, we recently established a simple method for quantifying the aversive response to high salt by using filter paper impregnated with sodium chloride and examined the aversive response to high salt in healthy subjects and CKD patients^11^. We found that salt taste recognition, as well as the aversive response to high salt, were reduced in CKD patients^11,12^, suggesting that the reduced aversive response to high salt concentration may make it more difficult to reduce salt intake, as they are unable to perceive high salt concentrations as unpleasant.

In addition, previous research has shown that simultaneous stimulation with different tastes can enhance or suppress the intensity of the perceived taste, a phenomenon known as “taste interactions”^13,14^. This could be relevant to our study as we aim to explore how sweet taste might affect the aversive response to high salt concentrations. We experience changes in taste perception when multiple taste stimuli are presented together in food or beverages, rather than individually. Studies of taste interactions have provided much information on how specific tastes interact with other taste stimuli^15^. For example, studies of two mixed tastes have shown that sweetness suppresses both sour^16–19^ and salt^19–22^ tastes, especially in medium to high concentrations or intensities of sweet taste^13^. These studies indicate the extent to which a particular taste is enhanced or suppressed by other taste stimulants, but little is known about what changes in taste aversiveness may result from such interactions.

In the present study, we focused on taste interactions as an external factor that may decrease aversiveness to high salt concentrations. We hypothesized that the combination of salt and sweet tastes would reduce the aversive response to high salt content in both healthy subjects and CKD patients. We investigated "the change in aversiveness to high salt concentrations caused by sweetening in both healthy subjects and CKD patients. We also examined whether the addition of sweetness changed the aversiveness to other tastes.

## Methods

### Participants

Healthy subjects were recruited from the general public between October 1, 2020 and March 31, 2022. Staff and students from our institution were recruited based on the general recruitment criteria, but only from departments other than our own. They were considered individuals with potential conflicts of interest. We recruited patients with CKD who were outpatients or inpatients at the Department of Nephrology, Kyoto Prefectural University Hospital between October 1, 2020 and March 31, 2022 as participants in this cross-sectional study.

Participants were required to be at least 20 years old and to provide informed consent to participate in the study. For healthy subjects, we checked the results of their annual health check-ups, which are widely conducted in Japan. Subjects were excluded if they had CKD, defined as abnormal urinalysis results and an estimated glomerular filtration rate (eGFR) < 60 ml/min/1.73m^2^, or if they had lifestyle-related diseases such as hypertension, diabetes mellitus, or hyperlipidemia, or had experienced an acute illness within the past 6 weeks. Subjects who were routinely taking any medications were also excluded. Among CKD patients, those with cognitive decline, an inability to consent, psychiatric illness, oral lesions (e.g., tongue cancer and oral candida), active cancer (including those who had undergone treatment in the past 5 years or were scheduled to receive systemic cancer treatment), active infections (requiring systemic antimicrobial agents), those unable to cooperate with taste testing, and patients who had COVID-19 at the time of the study or were diagnosed shortly after participation were also excluded. Additionally, other patients deemed inappropriate by the attending physician were excluded. To specifically assess the effect of adding sweet taste to other tastes, participants who had an aversion to sweet taste itself were excluded. The present study was conducted in accordance with the ethical guidelines of the Declaration of Helsinki and was approved by the Ethics Committee of Kyoto Prefectural University of Medicine (approval number ERB-C-2081). Written informed consent was obtained from all participants.

### Baseline demographics and clinical characteristics

Baseline data on the following confounding factors in CKD patients were obtained from medical records: age, sex, body mass index, comorbidities (diabetes, hypertension, dyslipidemia, and hyperuricemia), medications (renin-angiotensin system (RAS) inhibitors, diuretics, and calcium channel blockers). Laboratory tests were performed within 2 weeks of the taste test. Creatinine (Cr), albumin, and electrolytes (sodium, potassium, chloride, phosphorus, calcium, zinc, and copper) were measured using standard laboratory procedures. eGFR was calculated according to the following formula^23^: “194 × [age (years)] − 0.287 × [serum creatinine (mg/dl)] − 1.094 × [0.739 if female]”. Diabetes mellitus was defined as a history of glucose-lowering treatment or an HbA1c value > 6.5% according to the National Glycohemoglobin Standardization Program. Hypertension was defined as a history of antihypertensive treatment or systolic/diastolic blood pressure > 140/90 mmHg. Hyperlipidemia was defined as a history of lipid-lowering therapy or LDL cholesterol > 140 mg/dL. Hyperuricemia was defined as a history of treatment or a uric acid level > 7.0 mg/dL. Information on smoking, alcohol consumption, and use of dentures was collected using questionnaires completed by subjects at the time of taste testing. All baseline data on baseline characteristics for healthy subjects were obtained using questionnaires completed by subjects at the time of taste testing.

### Measurement of gustatory and aversion thresholds

Assessments of gustatory and aversion thresholds were performed as described previously^11,24^ using a taste substance–impregnated test strip (Taste Disc, Sanwa Kagaku Kenkyusho Co., Ltd.), a common tool in clinical practice for evaluating taste disorders. In brief, reagents were used at five concentrations: sucrose (0.3, 2.5, 10, 20, and 80%) for the sweet taste, tartaric acid (0.02, 0.2, 2, 4, and 8%) for the sour taste, and quinine hydrochloride (0.001, 0.02, 0.1, 0.5, and 4%) for the bitter taste. For the salt taste, we developed nine levels of NaCl (0.3, 0.6, 0.9, 1.2, 1.5, 2.0, 5, 10, and 20%) to examine taste thresholds at finer concentrations. To evaluate the effect of sweetness, 80% sucrose was added in equal amounts to each reagent for salt, sour, and bitter tastes. Following the previously established protocol^24^, participants placed filter paper with one drop of each of these reagents on their tongue in order from the lowest to highest concentration. They were first asked whether they perceived any taste. If they responded affirmatively, they were then asked to identify the taste and whether they found it unpleasant. We asked about “unpleasantness” instead of “preference” because we expected few participants to find flavored filter paper pleasant, unlike in food or beverage-based taste tests^25–27^. We defined the detection threshold for the lowest concentration sensed when participants tasted the reagents as the recognition threshold for the concentration that may be perceived as the taste type (salt, sweet, sour, and bitter tastes) and the aversion threshold as the concentration that was initially unpleasant. Furthermore, since filter paper itself may impart a taste, we placed a piece of neutral (unflavored) filter paper on the tongue as a control. Additionally, to minimize taste carryover, participants rinsed their mouths with water between different taste types.

### Statistical analysis

Statistical analyses were performed using JMP Pro 16 (SAS Institute Inc., Cary, NC, USA) to assess the significance of relationships between variables. Fisher’s exact test was used for nominal scales and the Wilcoxon test for continuous scales. A *p* value < 0.05 was considered to indicate a significant difference.

## Results

### Changes in the taste aversion threshold by adding sweetness in healthy subjects

**Fig. 1:**
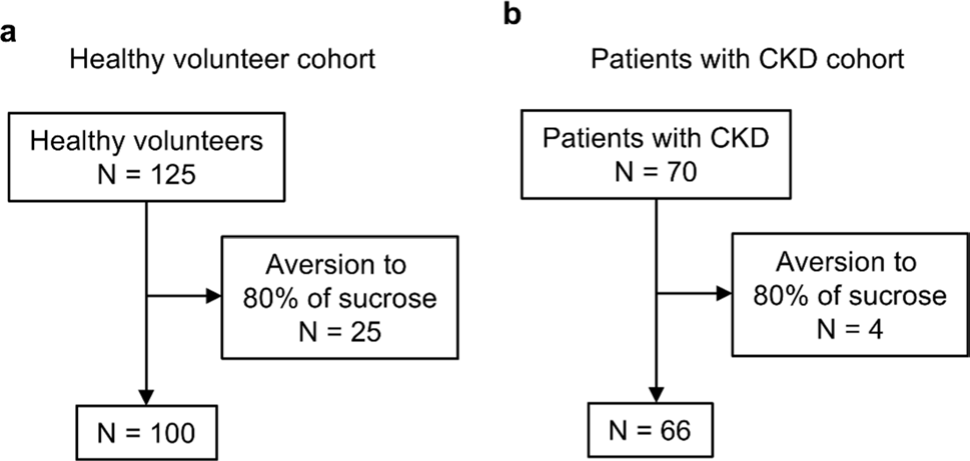
Subject selection. (**a**,**b**) The taste test was conducted on 100 healthy subjects out of 125, excluding 25 who disliked sweet taste (**a**), and on 66 CKD patients out of 70, excluding 4 who disliked sweet taste (**b**).

A taste test was performed on 125 healthy volunteers and 70 patients with CKD using nine concentrations of NaCl (0.3% to 20%) and five concentrations of sucrose, tartaric acid, and quinine hydrochloride. Participants tasted the solutions on filter paper with their tongues and defined the lowest concentration they found unpleasant (the aversion threshold). To assess the specific effect of adding sweetness to other tastes, participants who exhibited aversion to 80% sucrose alone were excluded from the original study population^11^. As a result, 100 healthy volunteers and 66 patients with CKD were included in the final analyses (Fig. 1).

**Fig. 2:**
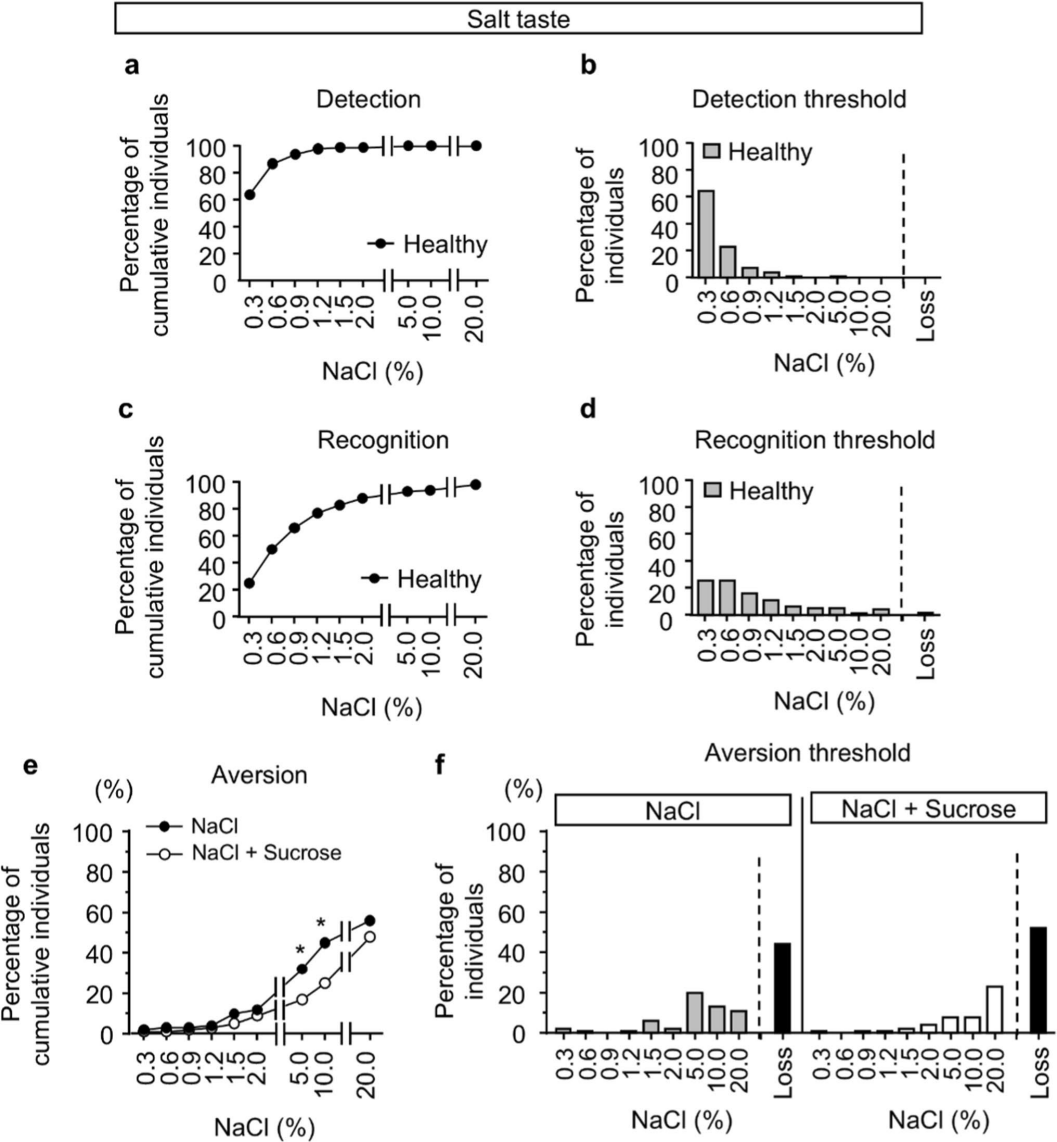
Detection, recognition, and aversion thresholds for NaCl in healthy volunteers. (**a**–**d**) Cumulative percentage of individuals responding to various concentrations of NaCl in detection (**a**) and recognition (**c**) in healthy volunteers. Percentage of individuals for various NaCl concentrations in detection (**b**) and recognition (**d**) in healthy volunteers. (**e**,**f**) Cumulative percentage (**e**) and individual percentage (f) of aversive responses to various concentrations of NaCl, with or without 20% sucrose in healthy volunteers. Participants who failed to detect, recognize, or show aversion even at the highest concentration of NaCl were categorized as “Loss.” Fisher’s exact test was used for the statistical analysis at each indicated concentration. *p < 0.05.

After excluding the participants who had an aversion to high concentrations of sucrose, the median age of the healthy participants was 38 years, and the proportion of women was 59% (Table 1). At first, the detection and recognition thresholds for salt taste were examined. For detection threshold, 64% and 87% of healthy participants could sense the reagents containing 0.3% and 0.6% salt, respectively (Fig. 2a,b). According to previous studies showing that the normal recognition threshold for salt taste is 0.6%^24,28^, 50% and 66% of the healthy participants recognized salt concentrations of 0.6% and 0.9%, respectively (Fig. 2c,d). No aversion to salt taste was observed at lower concentrations, but it became apparent at 5.0% NaCl and increased with concentration (Fig. 2e,f). However, while only 2.0% of participants failed to recognize 20% salt, 44% of healthy subjects showed no aversion even at 20% salt (Fig. 2c,e,f). The addition of sweet taste reduced aversion to salt, particularly at 5% and 10% NaCl. Aversion to these concentrations decreased from 32 and 45% to 17% and 25%, respectively, when sweetness was added (Figs. 2e,f).Furthermore, the proportion of healthy participants who showed no aversion to 20% salt increased from 44 to 52% with the addition of sweetness.

**Table 1:** Characteristics of healthy subjects.

	All (N = 100)	Change in aversion to NaCl		*p* value
		Yes (N = 38)	No (N = 62)	
Age	38.0 (30.0–49.0)	38.0 (30–49)	39.5 (29–50.5)	0.8063
Gender (Male/Female)	41/59	15/23	26/36	0.837
Body mass index (kg/m^2^)	21.1 (19.5–22.9)	21.0 (19.5–24.2)	21.1 (19.4–22.7)	0.994
Smoking (Yes/No)	11/89	2/36	9/53	0.1983
Drinking (Yes/No)	59/41	24/14	35/27	0.537
False teeth (Yes/No)	14/86	9/29	5/57	0.0388

Continuous variables are presented as medians (interquartile ranges).

*Fisher’s exact test was used for nominal scales and the Wilcoxon test for continuous scales.

**Fig. 3:**
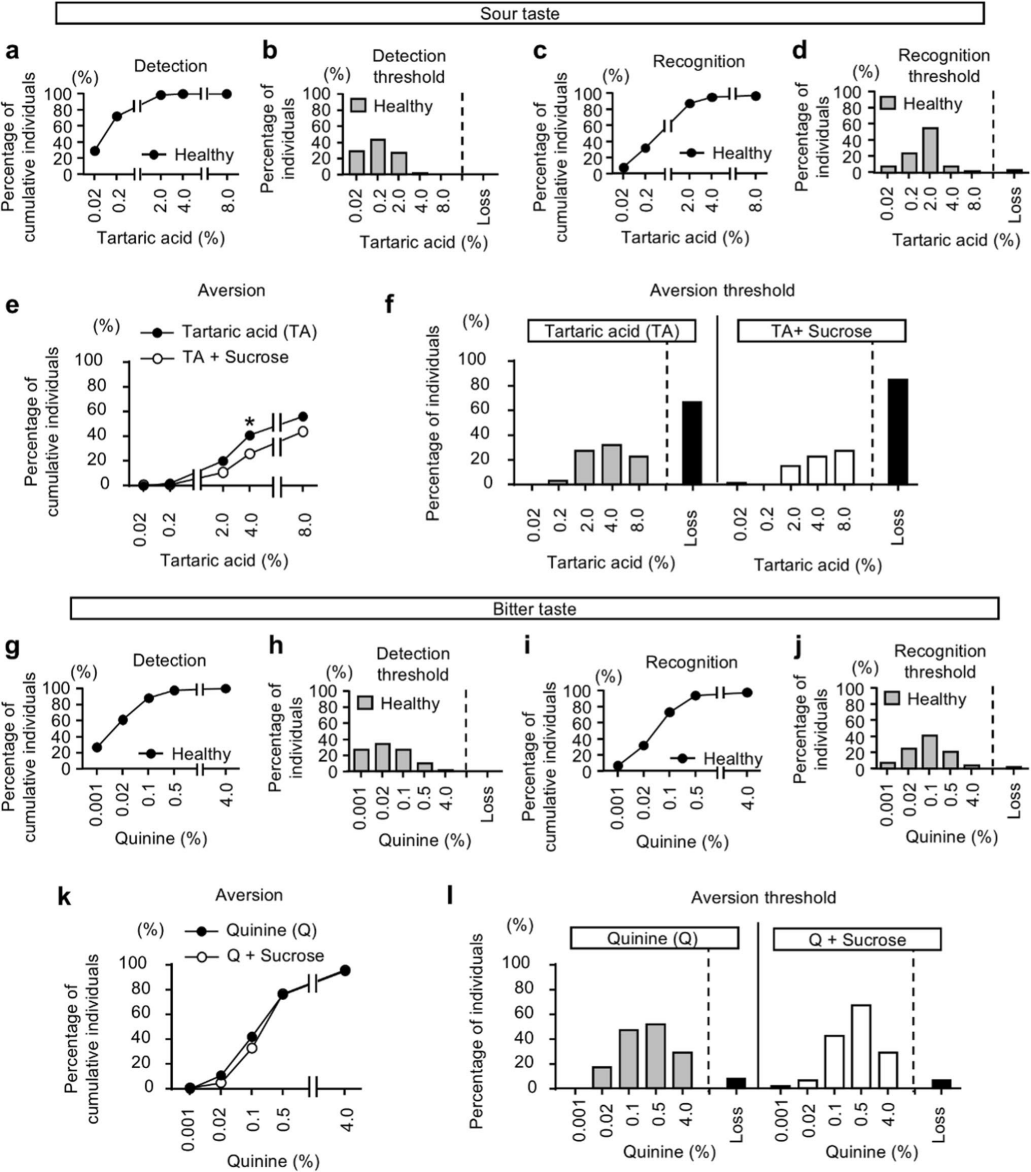
Detection and recognition thresholds for sour and bitter tastes in healthy volunteers. (**a**–**d**) Cumulative percentage of individuals responding to various concentrations of tartaric acid in detection (**a**) and recognition (**c**) in healthy volunteers. Percentage of individuals for various tartaric acid concentrations in detection (**b**) and recognition (**d**) in healthy volunteers. (**e**,**f**) Cumulative percentage (**e**) and individual percentage (**f**) of aversive responses to various concentrations of tartaric acid, with or without 20% sucrose in healthy volunteers. (**g**–**j**) Cumulative percentage of individuals responding to various concentrations of quinine in detection (**g**) and recognition (**i**) in healthy volunteers. Percentage of individuals for various quinine concentrations in detection (**h**) and recognition (**j**) in healthy volunteers. (**k**,**l**) Cumulative percentage (**k**) and individual percentage (**l**) of aversive responses to various concentrations of quinine, with or without 20% sucrose in healthy volunteers. Subjects unable to detect or recognize even the highest concentration of quinine were described as “Loss”. Fisher’s exact test was used for the statistical analysis at each indicated concentration. *p < 0.05.

For the detection of other tastes, approximately 90% of the test subjects were able to detect up to the third concentration out of five (Fig. 3a,b,g,h; Supplementary Figure S1a,b). For the recognition of other tastes, about 60% or more of the test subjects were able to recognize up to the third concentration out of five (Fig. 3c,d,i,j; Supplementary Figure S1c,d). To examine aversion to sour and bitter tastes, we conducted taste tests using increasing concentrations of each stimulant until participants reported the taste as unpleasant. Aversion to sour and bitter tastes increased in a concentration-dependent manner, with aversion to bitterness being more pronounced. Similar to salt taste, the addition of sweetness reduced aversion to sour taste, with a significant decrease observed at the 4% concentration. However, unlike sour and salt tastes, bitterness remained strongly aversive even after the addition of sweetness (Fig. 3e,f,k,l).

### Changes in the taste aversion threshold by adding sweetness in patients with CKD

**Fig. 4:**
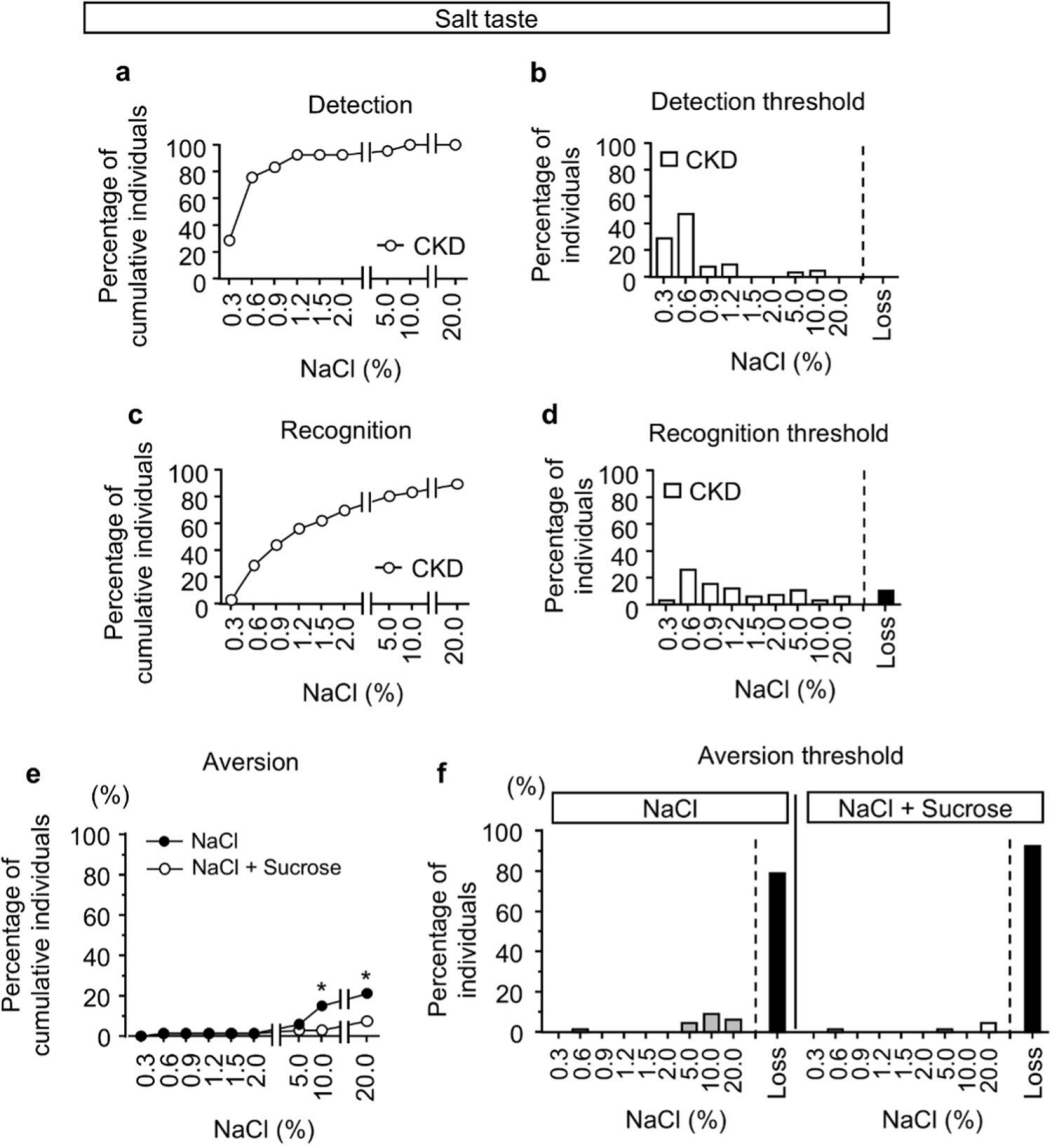
Detection, recognition and aversion thresholds for NaCl in CKD patients. (**a**–**d**) Cumulative percentage of individuals responding to various concentrations of NaCl in detection (**a**) and recognition (**c**) in CKD patients. Percentage of individuals for various NaCl concentrations in detection (**b**) and recognition (**d**) in CKD patients. (**e**,**f**) Cumulative percentage (**e**) and individual percentage (f) of aversive responses to various concentrations of NaCl, with or without 20% sucrose in CKD patients. Participants who failed to detect, recognize, or show aversion even at the highest concentration of NaCl were categorized as “Loss.” Fisher’s exact test was used for the statistical analysis at each indicated concentration. *p < 0.05.

We investigated whether adding a sweet taste would similarly reduce aversion to high salt tastes in patients with CKD. Excluding those who found the sweet taste itself aversive, patients with CKD were significantly older than healthy participants, with a median age of 66.5 years compared to that of the healthy group, and were more often male (59.1%) (Table 2). The median serum creatinine level was 2.22 mg/dL, and 16.7% of the patients had diabetes.　Regarding the detection threshold, 28.8% and 72.4% of CKD patients were able to detect salt at concentrations of 0.3% and 0.6%, respectively (Fig. 4a,b). For the recognition thresholds, only 28.8% and 43.9% of participants recognized salt at concentrations of 0.6% and 0.9%, respectively, and 10.6% of CKD patients did not recognize even the highest concentration of 20% NaCl (Fig. 4c,d).

**Table 2:** Characteristics of CKD patient.

	CKD (N = 66)	Change in aversion to NaCl		*p* value
		Yes (N = 12)	No (N = 54)	
Age	66.5 (54.8–76)	63 (47.3–76.5)	67.5(55–76)	0.6414
Gender (Male/Female)	39/27	3/9	36/18	0.0109
Body mass index (kg/m^2^)	23.3 (20.7–25.8)	21.4 (20.4–25.4)	23.8 (21.6–26.0)	0.2064
Smoking (Yes/No)	11/55	2/10	9/45	1
Drinking (Yes/No)	32/34	3/9	29/25	0.1104
False teeth (Yes/No)	22/44	2/10	20/34	0.3099
BUN(mg/dL)	31.9 (19.7–62.3)	49.15 (12.15–71.23)	31.6 (19.7–56.35)	0.9730
Creatinine (mg/dL)	2.22 (1.22–4.90)	3.97 (0.88–5.77)	2.05 (1.23–3.92)	0.5719
eGFR (ml/min/1.73m^2^)	24.5 (10.1–47.5)	14.25 (8.66–69.81)	27.43 (12.43–45.39)	0.6297
Diabetes (Yes/No)	11/55	2/10	9/45	1.0000
Hypertension (Yes/No)	53/13	10/2	43/11	1.0000
Hyperlipidemia (Yes/No)	34/32	6/6	28/26	1.0000
Hyperuricemia (Yes/No)	34/32	6/6	28/26	1.0000
Sodium (mmol/L)	139 (137.5–141)	141 (139.5–142)	139 (137–141)	0.1280
Potassium (mmol/L)	4.2 (3.95–4.45)	4.4 (3.9–4.5)	4.2 (4.0–4.4)	0.5133
Chloride (mmol/L)	105 (101.5–106)	106 (104–108)	104 (101–106)	0.0422
Phosphate (mg/dL)	3.7 (3.3–4.75)	4.4 (3.6–5.6)	3.6 (3.3–4.6)	0.0792
Calcium (mg/dL)	8.9 (8.4–9.3)	8.6 (8.0–9.2)	8.9 (8.4–9.3)	0.1296
Albumin (g/dL)	3.4 (3.05–3.9)	3.2 (2.8–3.3)	3.5 (3.1–3.9)	0.0902
Zinc (μg/dL)	67(60–81.8)	61 (46–74)	71 (62–87)	0.0915
Copper (μg/dL)	105.5(82.3–121.3)	111 (78–134)	104 (83–118)	0.2968

Continuous variables are presented as medians (interquartile ranges).

*Fisher’s exact test was used for nominal scales and the Wilcoxon test for continuous scales.

At 20% NaCl, 78.8% of CKD patients had completely lost aversion to the salty taste (Fig. 4e,f). To examine factors contributing to decreased aversion to salt taste, we divided CKD patients into two groups: one with and one without change in aversion to high salt by the addition of sweetness. Only 15.2% of CKD patients found 10% NaCl aversive. Among background characteristics, gender and serum chloride concentration were associated with change in the aversion to salt taste by the addition of sweetness (Table 2). Similar to the results in healthy participants, the addition of sweet taste significantly decreased aversion in CKD patients. The aversion rates at 10% and 20% NaCl decreased from 15.2% and 21.2% to 3.0% and 7.6%, respectively (Fig. 4e,f). These findings indicate that the addition of sweetness almost eliminated aversion to high salt concentrations.

**Fig. 5:**
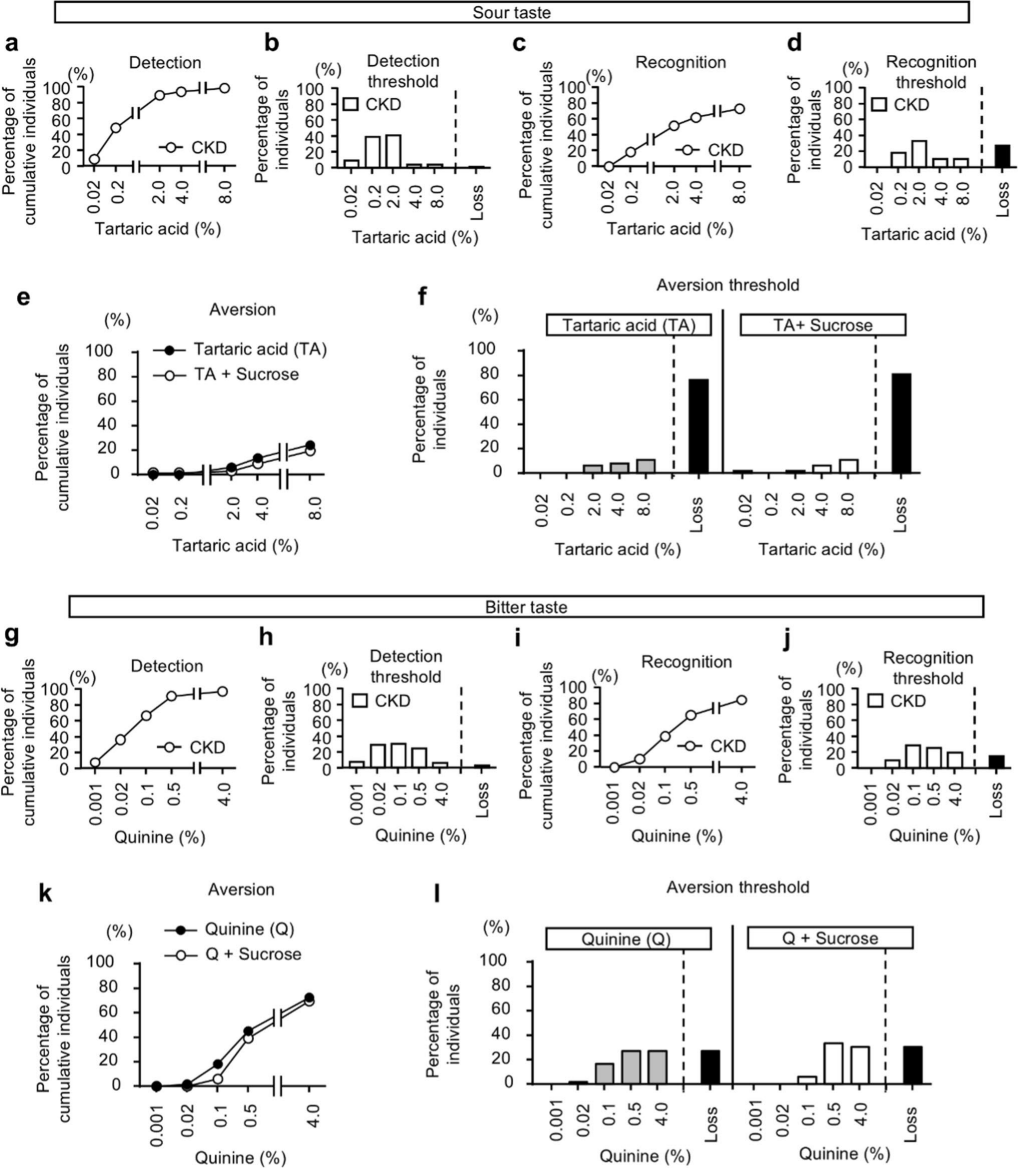
Detection and recognition thresholds for sour and bitter tastes in CKD patients. (**a**–**d**) Cumulative percentage of individuals responding to various concentrations of tartaric acid in detection (**a**) and recognition (**c**) in CKD patients. Percentage of individuals for various tartaric acid concentrations in detection (b) and recognition (**d**) in CKD patients. (**e**,**f**) Cumulative percentage (**e**) and individual percentage (**f**) of aversive responses to various concentrations of tartaric acid, with or without 20% sucrose in CKD patients. (**g**–**j**) Cumulative percentage of individuals responding to various concentrations of quinine in detection (**g**) and recognition (i) in CKD patients. Percentage of individuals for various quinine concentrations in detection (h) and recognition (**j**) in CKD patients. (**k**,**l**) Cumulative percentage (**k**) and individual percentage (**l**) of aversive responses to various concentrations of quinine, with or without 20% sucrose in CKD patients. Subjects unable to detect or recognize even the highest concentration of quinine were described as “Loss”. Fisher’s exact test was used for the statistical analysis at each indicated concentration. *p < 0.05.

For the detection of other tastes, 79.7%, 85.6%, and 63.8% of CKD patients were able to detect up to the third concentration in the series of five concentrations for sweet, sour, and bitter tastes, respectively, which were lower than the corresponding values for healthy subjects (Fig. 5a,b,g,h; Supplementary Figure S1e,f.). For the recognition of these tastes, only 50.7%, 49.3%, and 37.7% of CKD patients were able to recognize up to the third concentration for sweet, sour, and bitter tastes, respectively, which were markedly lower than those of healthy subjects (Fig. 5c,d,i,j; Supplementary Figure S1g,h). Notably, 27.3% and 15.2% of CKD patients were unable to recognize even the highest concentrations of sour and bitter tastes, respectively (Fig. 5d,j).

Aversion to sour and bitter tastes also decreased in CKD patients, with 77.8% and 27.3% of patients completely losing their aversion, respectively (Fig. 5e,f,k,l). Unlike the results observed in healthy subjects, the aversion to high concentrations of tartaric acid was not affected by the addition of sweet taste (Fig. 5e,f). For bitter tastes, the aversion to high concentrations of quinine remained unchanged by the addition of sweet taste, similar to the findings in healthy subjects (Fig. 5k,l). The percentages of complete loss of aversion to sour and bitter tastes after adding sweet taste were 80.3% and 30.3%, respectively, and the changes compared to when each taste was tested alone were minimal (Fig. 5f,l).

## Discussion

The sense of taste consists of five fundamental tastes: salty, sweet, sour, bitter, and umami, with mammals commonly preferring sweet and umami, while disliking sour and bitter tastes^29^. Regarding salty taste, animal experiments have shown two opposite responses to salt: attraction to low concentrations and aversion to high concentrations^10^. Recently, we developed a simple and quantitative method to evaluate aversive responses to high concentrations of taste stimuli in humans, and showed that CKD patients have a reduced aversion to high concentrations of salt^11^. However, when we consume food or drink, we do not experience a single taste in isolation, but instead perceive the taste of food as a combination of several flavors. Therefore, it is important to assess how multiple taste stimuli interact with one another. In this study, we investigated how adding sweet taste, the most palatable flavor, affects aversive responses to various tastes in healthy subjects and CKD patients. The aversive response to high concentrations of salt was attenuated by the addition of sweet taste. In particular, while the aversion to high concentration of salt is further reduced in CKD patients, the adding sweetness almost completely eliminated its aversive response. These results suggest that it may be difficult to promote salt restriction by relying on aversive responses to high salt concentrations in the context of a sweetened diet.

It has long been recognized that mammals tend to crave salt, a phenomenon called “salt hunger”. Regarding its physiological role, as early as 1884 Maeley wrote: “The ‘universal existence of an appetite for salt surely indicates that the substance serves more important functions than that of merely gratifying the palate’^30^. NaCl is the most critical determinant of body fluid volume, and it is reasonable that terrestrial organisms prefer NaCl to maintain proper hydration and electrolyte balance. However, as shown in our human studies and previous animal experiments have shown, mammals exhibit an aversion to high salt concentrations^10–12^. In fact, in natural environments, mammals consume appropriate amounts of salt, whereas only humans have developed the habit of consuming salt in excess of physiological needs^31,32^. This raises the question: why do mammals avoid high concentrations of salt? NaCl is not only the most important determinant of extracellular fluid volume, but also the primary determinant of serum osmolality. Therefore, it is likely that organisms attempt to avoid ingesting excessive NaCl because it becomes difficult to distinguish between a surplus of NaCl and a deficit of free water. There are no studies quantifying taste interactions in animals, and the mechanisms underlying sweet taste-mediated alleviation of salt aversion—such as alterations in taste receptors and physiological processes—remain unclear. However, we presume that when NaCl is consumed alongside glucose, an important nutritional source, animals may prioritize calorie intake over the aversive response to salt^19^.

While overall taste sensitivity are reduced in elderly individuals^33^ and CKD patients^24,26,34–37^, studies examining the effects of disease on taste interactions remain limited. A previous study in elderly individuals showed that taste interactions are attenuated compared to those in younger individuals^22^. In our study, not only the recognition of salt taste but also the aversion to high salt concentrations was attenuated in CKD patients, and this aversive response was almost completely abolished by the addition of sweet taste. These findings suggest that it is extremely difficult for CKD patients to regulate salt intake based on salt taste perception. Since few patients exhibited an aversive response even to high salt concentrations alone, the mechanisms underlying the attenuation of salt aversion by sweet taste remain unclear. Moreover, little is known about the molecular mechanisms of taste interaction in animal studies, especially under disease conditions, highlighting the need for future research.

Regarding other tastes, the aversive response to sour taste was mitigated by the addition of sweetness in both healthy individuals and CKD patients, similar to what was observed with salt taste. This can be explained by previous findings showing that sweetness suppresses the perceived intensity of sourness^38^, thereby attenuating the aversive response to high concentrations of sour taste. In addition, since organic acids such as acetic acid are recognized as important energy sources^39,40^, it is presumed that when consumed concurrently with glucose, the organism may prioritize energy intake over the aversive response to sour taste. On the other hand, though previous reports showed that sweetness has been shown to suppress bitterness intensity^19^, in our study, the addition of sweetness did not alter the aversive response to bitter taste in either healthy individuals or CKD patients. The reason for this discrepancy remains unclear. However, it is believed that bitterness serves to signal the presence of poisons^41^ Thus, the instinctive drive to avoid bitter taste may override the appeal of the sweet substance, even when it serves as a valuable energy source.

This study has several limitations. First, we investigated the effect of high sucrose concentrations on aversive responses, but the effects of lower sucrose concentrations remain unclear. Second, although sweetening may reduce salt aversion, it is uncertain whether this leads to increased salt intake, which could reduce resistance to high salt concentrations. Third, the backgrounds of CKD patients differed from those of healthy subjects, with various confounding factors such as age and gender. Therefore, the effects of renal dysfunction on responses to different taste stimuli remain unclear. Fourth, due to the small number of CKD patients who initially showed aversion to salty and sour tastes, changes in aversive responses with the addition of sweetness may have been underestimated. Finally, the small number of CKD patients who showed an aversive response to high salt prevented us from identifying the factors responsible for the change in response when sweet taste was added. Further studies with a larger sample size are warranted.

In conclusion, this study demonstrated that adding sweetness reduces the aversive response to high salt concentrations in both healthy subjects and CKD patients. It is possible that adding sweetness to salt may have increased salt intake by reducing the avoidance of high salt concentrations. Therefore, it is extremely difficult to encourage salt reduction based on patients’ reduced sensitivity to saltiness. In addition, CKD patients often have impaired taste perception, making it difficult for them to sense high salt concentrations. As the number of patients with lifestyle-related diseases increases worldwide, appropriate dietary intake, including salt reduction, will become increasingly crucial in the future. To enhance the perception of saltiness in food, it is crucial to consider taste interactions. Enhancing the perception of saltiness by reducing sweetness may be an effective strategy for reducing overall salt intake.

## Electronic supplementary material

Below is the link to the electronic supplementary material.

Supplementary Material 1

## Data Availability

The data that support the findings of this study are available on request to the corresponding author, TK. The original data can be provided after the approval of the Ethics Committee of Kyoto Prefectural University of Medicine.
