# Supplementary material for: The addition of sweetness reduces aversion to high salt concentrations in patients with chronic kidney disease
Source: Sci Rep. 2025 Jul 7;15:24322. doi: 10.1038/s41598-025-09602-x (PMC12234979; doi:10.1038/s41598-025-09602-x)
Supplement: Supplementary file 1 — Supplementary Material 1 [file 41598_2025_9602_MOESM1_ESM.docx]

Supplementary Information

**The addition of sweetness reduces aversion to high salt concentrations in patients with chronic kidney disease**

Natsuko Okuno-Ozeki, Yusuke Kohama, Hiromu Taguchi, Yuka Kawate, Keiichi Tamagaki, Takuya Taniguchi, Takashi Hirao, Satoaki Matoba, and Tetsuro Kusaba

**
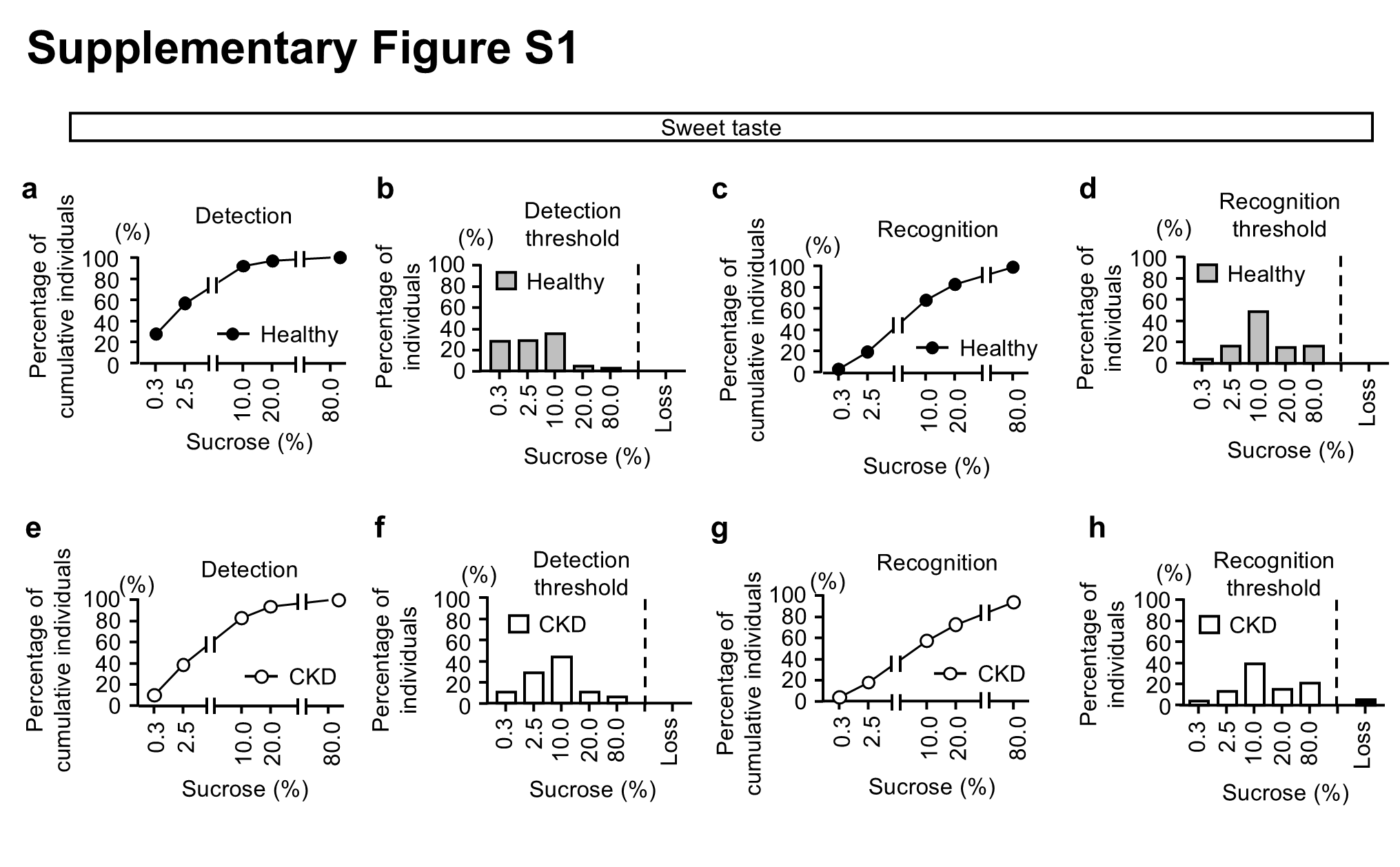
**

**Supplementary Figure S1. Detection and recognition thresholds for sucrose in healthy volunteers and patients with CKD**

(a)-(d) Percentage of cumulative individuals for various concentrations of sucrose for the detection (a) and recognition (c) in healthy volunteers. Percentage of individuals for various sucrose concentrations in detection (b) and recognition (d) in healthy volunteers. (e)-(h) Percentage of cumulative individuals for various concentrations of sucrose for the detection (e) and recognition (g) in patients with CKD. Percentage of individuals for various sucrose concentrations in detection (f) and recognition (h) in patients with CKD. Subjects unable to detect or recognize even the highest concentration of sucrose were described as “Loss”.
